# *NIBAN1*, Exploring its Roles in Cell Survival Under Stress Context

**DOI:** 10.3389/fcell.2022.867003

**Published:** 2022-04-19

**Authors:** Paula Diana, Gianna Maria Griz Carvalheira

**Affiliations:** Division of Genetics, Department of Morphology and Genetics, Universidade Federal de São Paulo, São Paulo, Brazil

**Keywords:** *NIBAN1*, *FAM129A*, diseases, cancer, cell microenvironment, autophagy, apoptosis

## Abstract

Cell survival must quickly activate specific mechanisms that enable to detect changes in the cellular microenvironment. The impact of these cell alteration has direct consequences on cellular homeostasis. Cellular stress, as well as its regulation and implication, has been studied in different pathologies. In this sense, the alteration in *NIBAN1* expression seems to act in response to different cellular disturbances. Over the years, the knowledge of *NIBAN1* functions has improved, demonstrating its important cell roles, favoring the cell survival under stress context. In response to the disturbances, *NIBAN1* seems to be involved in the decision-making process between cell survival and death. The increase in *NIBAN1* expression has been related to cellular mechanisms that seek to minimize the damage caused to cellular homeostasis. In this review, the main biological insights attributed to the *NIBAN1* gene in different cellular contexts and its role as a mediator of cellular stress are discussed.

## Introduction

Cellular machinery uses numerous molecular mechanisms to adapt to microenvironment, providing evolutionary success and cell survival. Biological processes associated with stress responses play significant roles in normal development and homeostasis. These biological mechanisms of stress adaptation contribute to formidable cellular resilience. However, these same biological events can also contribute to cellular degeneration over time, invariably culminating in aging and/or disease (Fuchs and Steller, 2015). Among the types of cellular disturbances, we can mention: DNA damage agents, which activate specific repair pathways (Chang et al., 2017; Galluzzi et al., 2018), heat shock or toxin-causing agents, which activate the unfolded protein response (UPR) in the endoplasmic reticulum (ER) and mitochondria (Galluzzi et al., 2018; Hetz and Papa, 2018), hypoxia, respiratory poisons and xenobiotics, which cause mitochondrial stress (Galluzzi et al., 2018; Suomalainen and Battersby, 2018), nutrient deprivation, which activates autophagy (Galluzzi et al., 2017; Galluzzi et al., 2018), and infectious agents, leading to multiples stress responses (Cao, 2016; Galluzzi et al., 2018). To combat these adverse conditions, cells activate fast and transient programs that adjust both RNA and protein synthesis, cytoskeletal and membrane integrity, as well as cell homeostasis (Richter et al., 2010; Vihervaara et al., 2018). In each cell, organelles employ autonomous signaling strategies, which detect and communicate dangerous conditions to both cytosol and nucleus, inducing specific and global transcriptional responses (Walter and Ron, 2011; Vihervaara et al., 2018). This communication is achieved by multiple mechanisms, including (but not limited to) changes in the shape of stressed cells and their connections with the microenvironment, the exposure of specific molecules on its surface, and the active or passive release of bioactive factors, such as ions, small metabolites, intracellular proteins, cytokines, or microvesicles (Galluzzi et al., 2018). Multiple potentially harmful perturbations of the intracellular or extracellular microenvironment can be successfully managed by mammalian cells after activating stress responses that preserve cellular functions and repair macromolecular damage (Galluzzi et al., 2018).

Understanding the molecular mechanism as well as the change in the gene expression profile, in response to cellular stress, had been an important issue for the Oncology (Chen and Cubillos-Ruiz, 2021). In this cellular context, the analysis of the gene expression, in response to stress, has been one of the methodological strategies. Many genes have been studied as biomarkers for various diseases and cancer. *NIBAN1* is one of these gene, whose expression is unregulated in many cancer and diseases, such as renal cancer (Majima et al., 2000; Adachi et al., 2004; Hino, 2004; Sun et al., 2007; Feng et al., 2019), lung cancer (Ji et al., 2012; Zhang N. et al., 2019), head and neck carcinoma (Ito et al., 2010), thyroid cancer (Cerutti et al., 2004; Maciel et al., 2005; Cerutti et al., 2006; Matsumoto et al., 2006; Cerutti, 2011; Patel et al., 2011; Carvalheira et al., 2013; Carvalheira et al., 2015; Nozima et al., 2019), gynecologic cancers (Yuki et al., 2015; Evstafieva et al., 2018; Salgado-Albarrán et al., 2019; Wang et al., 2020; Chen et al., 2021), prostate cancer (Shaw et al., 2016; Thomas et al., 2016; Pällmann et al., 2019), brain cancer (Miller et al., 2011; Qaisiya et al., 2017), bladder cancer (Zhu et al., 2018; Jiang et al., 2020) and colorectal cancer (Tan et al., 2021; Wang et al., 2021). The *NIBAN1* expression has also been observed in some diseases such as, renal interstitial fibrosis (Liu et al., 2014; Tang et al., 2019; Tang et al., 2021), in asthmatic patients treated with glucocorticoids (Yick et al., 2013; Yick et al., 2014), arterial diseases (Chen et al., 2019), vasomotor dysfunction (Luo et al., 2017; Yim et al., 2020), liver diseases (Kannangai et al., 2005), and pancreatic diseases (Zhang K. et al., 2019). This review analyzes the-state-of-the-art regarding to *NIBAN1* gene and its biological role in response to cell stress.

## The Niban Apoptosis Regulator 1 (*NIBAN1*)

The *NIBAN1* gene encodes a protein that belongs to the FAM129 family (family with sequence similarity 129). This protein family is encoded by three genes: *NIBAN1*, located at 1q25.3 (aliases: *C1orf24*; *FAM129A*; *NIBAN*; *GIG39*); *NIBAN2*, located at 9q34.11 (aliases: *OC58*; *MEG-3*; *C9orf88*; *FAM129B*; *MINERVA*; *bA356B19.6*), and *NIBAN3*, located at 19p13.11 (aliases: *BCNP1*; *FAM129C*). These genes share a pleckstrin homology (PH) domain (PH) in their N-terminal region. The PH domain seems to be involved in signaling, cytoskeleton organization, membrane trafficking, and phospholipid processing (Lenoir et al., 2015). Amino acids sequence analysis of the FAM129 family members revealed that they share a common PH domain on their N-terminal region with high sequence similarity of 57% between *NIBAN1* and *NIBAN2*, and 27% between *NIBAN2* and *NIBAN3* (Old et al., 2009).

These genes have significant cellular functions for many diseases and carcinogenic processes. As mentioned before, the *NIBAN1* protein is highly expressed in several diseases and cancer cells, and may play an important role in cell maintenance, such as cell stress (Adachi et al., 2004; Sun et al., 2007; Ji et al., 2012; Qaisiya et al., 2017; Nozima et al., 2019; Pällmann et al., 2019; Yim et al., 2020), autophagy (Qaisiya et al., 2017; Nozima et al., 2019), and others survival biological processes (Table 1). *NIBAN2* gene seems to be involved in ERK pathway, at least, in human melanoma cells (Chen et al., 2011). On the other hand, *NIBAN3* encodes the membrane protein detected in B lymphocytes from chronic lymphocytic leukemia patients that seems to be involved in regulates B-cell receptor (BCR) signaling and B-cell apoptosis (Boyd et al., 2003).

**TABLE 1:** The biological effects of *NIBAN1* upregulation in human cancer.

Biological effect	Cancer type	Genomic and molecular context	References
Cell stress	Kidney cancer	Mutations Tsc1/Tsc2; mTOR pathway; endoplasmic reticulum stress; translation process; knockout of NIBAN1; knockdown of NIBAN1	Majima et al. (2000)
			Adachi et al. (2004)
			Sun et al. (2007)
	Thyroid cancer	Altered mitochondrial functions; cell stress; knockdown by miR-106b	Matsumoto et al. (2006)
			Carvalheira et al. (2013)
			Carvalheira et al. (2015)
	Prostate cancer	Regulation by AR; regulation by ATF4; eIF2α phosphorylation via PERK; endoplasmic reticulum stress; translation process	Shaw et al. (2016)
			Thomas et al. (2016)
			Pällmann et al. (2019)
	Colon cancer	Regulation by ATF4	Evstafieva et al. (2018)
Cell migration and proliferation	Thyroid cancer	Knockdown by miR-106b	Carvalheira et al. (2015)
	Colon cancer	Overexpression of zinc-finger protein 777	Yuki et al. (2015)
	Kidney cancer	Knockdown by miR-4521; TIMP-1/MMP2/MMP9	Feng et al. (2019)
	Lung cancer	FAK signaling pathway	Zhang N. et al. (2019)
Cell apoptosis	Lung cancer	Interaction with NPM, MDM2 and p53 degradation	Ji et al. (2012)
	Thyroid cancer	Knockdown by miR-106b	Carvalheira et al. (2015)
	Kidney cancer	MDM2/p53/Bcl2/Bax pathway	Feng et al. (2019)
Cell autophagy	Nerve cells cancer	Unconjugated bilirubin neurotoxicity, oxidative stress	Qaisiya et al. (2017)
	Thyroid cancer	Oncogene-dependent; mutation RET/PTC1; AKT/mTOR/p70S6K	Nozima et al. (2019)
Imune microenvironment	Colon cancer	CCDC69, CLMP, FAM110B, GUCY1B3, PALLD, PLEKHO1, STY11; tumor mutational burden	Wang et al. (2020)
	Colorectal cancer	Overexpression BCL2, PMAIP1 and RPS6; immune infiltration	Tan et al. (2021)

The *NIBAN1* gene was described in 2000 by Majima et al. (2000) in renal carcinoma, both in murine and human cell lines. This gene was called “Niban” (“second” in Japanese), as it is the second gene found by Majima group, after Erc (expressed in renal carcinoma). According to the Gene Nomenclature Committee, from 2019, an official nomenclature for this gene was stablished and would be *NIBAN1* (Braschi et al., 2019).

The *NIBAN1* messenger RNA (mRNA) identified by Majima et al. (2000) presented expression in normal brain, lung, spleen, and skeletal muscle from Wistar rats. In normal human tissues, strong *NIBAN1* expression was found in heart, skeletal muscle, pancreas, white blood cells, and prostate, whereas it presented moderate expression in colon and spleen. They also demonstrated that *NIBAN1* expression was dramatically increased in the early stage of renal carcinogenesis and that *NIBAN1* had a conserved structure between humans and rats. The absence of expression in normal rat/human kidneys and strong expression in tumor suggests an inverse relationship between *NIBAN1* expression and progression of renal carcinogenesis (Majima et al., 2000). Although the expression of *NIBAN1* has been described more than 20 years ago, its functions are not elucidated yet.

## *NIBAN1*: Structure and Expression

The structure of *NIBAN1* gene is located at reverse strand (antisense) of the 1q25.3 region, and has 184,784 kb, according to the coordinates 184.790.724–184.974.508 (GRCh38.p13, Figure 1). This gene has a predicted promoter region containing 9,797 bp, 16 exons, and presents five transcriptional variants (Genome Browser, GRChHg38.p13). Using gene expression data obtained from the Atlas of Human Proteins database (HPA, version: 20.1), under basal conditions, it is observed that smooth muscle, urinary bladder, and salivary gland have high transcriptional levels of *NIBAN1*, while protein levels are high in breast, bronchus, endometrium, Fallopian tube, liver, pancreas, and tonsil (Uhlén et al., 2005; Uhlén et al., 2015; Uhlen et al., 2017).

**FIGURE 1:**
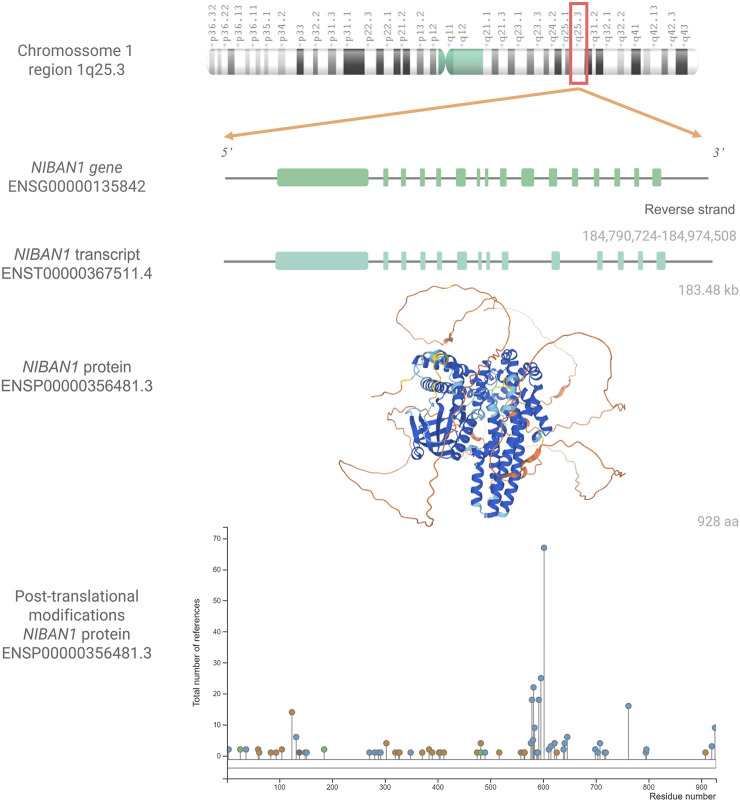
*NIBAN1*: gene, transcript, protein and post-tranlational modifications. Chromosome 1q25.3 showing the *NIBAN1* gene (ENSG00000135842) in the region 184,790,724-184,974,580, with its 16 exons, followed by the transcribed variant ENST00000367511.4 with 14 exons (Ensembl Genome Browser, GRCh38.p13) as well as the three-dimensional predicted NIBAN1 protein structure (ENSP00000356481.3). Structuring cores indicate the degree of reliability of the prediction. Dark Blue = Very High, Light Blue = Confident, Yellow = Low, and Orange = Very Low. Image available from AlphaFold, https://alphafold.ebi.ac.uk/entry/Q9BZQ8 (version: 2.1.0) (Jumper et al., 2021; Varadi et al., 2022). Graph representing the post-translational modifications in the respective residues of the NIBAN1 (ENSP00000356481.3). Phosphorylation marks are represented by blue circles; acetylation, by green circles; ubiquitination, by orange circles, and other types of marks by gray circles. Image available from PhosphoSitePlus®, https://www.phosphosite.org/uniprotAccAction?id=Q9BZQ8 (version: 6.5.9.3) (Hornbeck et al., 2015).

In humans, the NIBAN1 protein (ENSP00000356481.3, Figure 1) has 928 amino acids, and its location seems to be in some cellular compartments, such as cytoplasmic (Adachi et al., 2004; Cerutti et al., 2006; Matsumoto et al., 2006; Ito et al., 2010; Zhang N. et al., 2019), membranous (Uhlén et al., 2005; Uhlén et al., 2015; Uhlen et al., 2017), associated with both ER and plasma membrane, at least in prostate cancer (Pällmann et al., 2019), and nucleus (Adachi et al., 2004; Uhlen et al., 2017). It has been shown that under homeostasis conditions, the NIBAN1 protein has a 130 kDa molecular weight. However, under stress conditions, such as heat shock, oxidative stress, hypertonic stress, and endotoxin (lipopolysaccharide) administration, NIBAN1 could be observed in two sizes, 70 kDa and 130 kDa (Adachi et al., 2004). Note that, NIBAN1 isoform had been predicted, containing around 100 aa (ENSP00000414039.1, GRCh38.p13). However, this isoform did not have yet been described *in vivo* associated with NIBAN1 expression. Under stressful conditions it is suggested that the NIBAN1 moves from the cytoplasm to the nucleus. It is important to address that NIBAN1 has a dnaJ domain in its polypeptide sequence. This domain is found in members of the heat shock protein family, up-regulated under stress conditions (Adachi et al., 2004). Thus, possibly the presence of 70 kDa NIBAN1 may be a consequence of the cellular stress.

It has been described that NIBAN1 protein can undergo several post-translational modifications, being the phosphorylations marker the most common one (Figure 1) (Sun et al., 2007; Ji et al., 2012; Luo et al., 2017; Yim et al., 2020). Ji et al. (2012) suggested that AKT seems to be responsible for the NIBAN1 phosphorylation. According to predictions from the PhosphoSitePlus database (version: 6.5.9.3), NIBAN1 phosphorylation seems to occur at different serine, tyrosine, and threonine residues. On the other hand, acetylation and ubiquitination can occur in lysine residues (Hornbeck et al., 2015).

As mentioned above, *NIBAN1* expression occurs in different types of tumors (Majima et al., 2000; Adachi et al., 2004; Cerutti et al., 2004; Sun et al., 2007; Ji et al., 2012; Shaw et al., 2016; Qaisiya et al., 2017; Evstafieva et al., 2018; Zhu et al., 2018; Feng et al., 2019; Wang et al., 2021). Data from The Cancer Genome Atlas (TCGA) integrated into the Pathology Atlas (The Pathology Atlas, version: 20.1) (Uhlén et al., 2005; Uhlén et al., 2015; Uhlen et al., 2017) have shown increased expression of *NIBAN1* in several types of cancer, many has been described in both early carcinogenesis process (Adachi et al., 2004; Matsumoto et al., 2006; Ito et al., 2010) and associated with stress response (Adachi et al., 2004; Sun et al., 2007; Ji et al., 2012; Luo et al., 2017; Qaisiya et al., 2017; Nozima et al., 2019; Pällmann et al., 2019; Yim et al., 2020). Under stress conditions, *NIBAN1* seems to play an important role in the regulation of apoptosis, preventing cell death and allowing tumor progression (Sun et al., 2007; Ji et al., 2012; Nozima et al., 2019; Pällmann et al., 2019). However, the molecular mechanisms underlining *NIBAN1* expression as well as its biological role in carcinogenesis are not elucidated yet.

As stated earlier, the *NIBAN1* was overexpressed in early renal carcinogenesis in human and rats (Majima et al., 2000). The dysregulated expression of *NIBAN1* has also been described in head and neck squamous cell carcinomas (HNSCCs), as well as in squamous dysplasia of the mucosa of the head and neck (Ito et al., 2010). In these lesions, the *NIBAN1* expression has been observed in mild dysplasia, gradually increasing during carcinogenesis. This data suggests that *NIBAN1* overexpression may be an important process in HNSCCs initiating carcinogenesis and maintenance of this tumor progression (Ito et al., 2010). In thyroid cancer, increased *NIBAN1* expression has been described in different tumors subtypes: microcarcinomas; papillary carcinomas; follicular carcinomas; metastases, and tumors with oxyphilic cytoplasm, such as Hürthle cell carcinoma, and some oxyphilic cells, originating from Hashimoto’s Thyroiditis (Cerutti et al., 2004; Maciel et al., 2005; Cerutti et al., 2006; Matsumoto et al., 2006; Cerutti, 2011; Patel et al., 2011; Carvalheira et al., 2013; Carvalheira et al., 2015; Nozima et al., 2019). Due to the presence of *NIBAN1* in many histological thyroid tumors, it has been suggested that this gene may be overexpressed from the early stage of carcinogenesis and remain expressed during neoplastic progression (Matsumoto et al., 2006). Also, in lung cancer, *NIBAN1* overexpression was associated with advanced staging, lymph node metastasis, and poor survival rates (Zhang N. et al., 2019). Corroborating these data, in ovarian cancer, *NIBAN1* overexpression was related to poor prognosis, chemoresistance, and metastasis (Salgado-Albarrán et al., 2019).

As aforementioned, *NIBAN1* expression has been detected in Hashimoto’s thyroiditis. In this type of lesions, thyrocytes are under both chronic inflammation and stress condition, due to oxidative damage, presenting many modified mitochondria with oxyphilic cytoplasm (Ralli et al., 2020). As a result of the cellular characteristics of these neoplasia, it has been suggested that *NIBAN1* may be closely associated with altered mitochondrial functions in pre-neoplastic conditions and in oxyphilic tumors (Matsumoto et al., 2006). In prostate cancer, *NIBAN1* expression is regulated by androgen receptor (AR) (Shaw et al., 2016). It had been demonstrated the existence of a strong AR transcription factor binding site at the *NIBAN1 locus*. These data suggest that *NIBAN1* may be regulated by androgens, and its expression may increase in hormone-sensitive tissues (Thomas et al., 2016). CBFβ and RUNX2 are others transcription factors that activate *NIBAN1* expression in colon cancer (Wang et al., 2021). On the other hand, in ovarian cancer, it had been observed that the BORIS (Brother of the Regulator of Imprinted Sites) transcription factor downregulates both *AR* and *NIBAN1* expression (Salgado-Albarrán et al., 2019). All these data together suggest that *NIBAN1* may be a biomarker or even a therapeutic target (Adachi et al., 2004; Cerutti et al., 2004; Cerutti et al., 2006; Matsumoto et al., 2006; Ito et al., 2010; Carvalheira et al., 2013; Zhang N. et al., 2019).

## *NIBAN1* Functions

### Cell Stress

As mentioned earlier, *NIBAN1* overexpression was found in early renal carcinogenesis in human and Eker rats (Majima et al., 2000), as well as in many neoplastic lesions. These findings suggest that its expression is commonly induced in the early stage of carcinogenesis, regardless of its genetic context. To confirm that *NIBAN1* expression was associated with the genetic context, it was demonstrated in Eker rats, which have mutations in the *Tsc1* and *Tsc2* genes, that the expression of *NIBAN1* seems to act independently of the mTOR pathway (Majima et al., 2000; Adachi et al., 2004). The group also demonstrated that NIBAN1 presented itself with 130 and 70 KDa, in response to different stressors (heat shock, oxidative stress, hypertonic stress, and endotoxin), suggesting that this protein could undergo post-translational modifications (Adachi et al., 2004). On the other hand, Sun et al. (2007) demonstrated that *NIBAN1* expression seems to be induced by ER stress. They also observed that, in knockout mouse for *NIBAN1*, the phosphorylation in both p70 ribosomal kinase S6 subunit (S6K) and eukaryotic initiation translation factor 4E (4E-BP) was reduced. These findings suggest that *NIBAN1* may be involved with phosphorylation of S6K and 4E-EP in response to ER stress, which are significant components of the translation process. Moreover, they have also shown that reduced phosphorylation of these proteins modulated cell death signaling (Sun et al., 2007).

**FIGURE 2:**
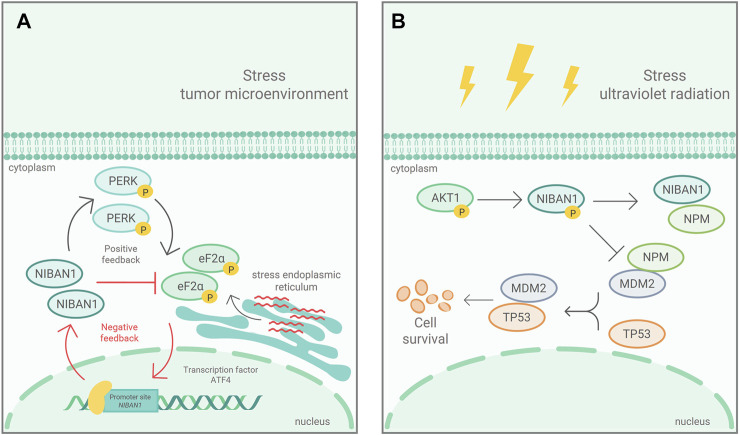
Mechanism of *NIBAN1* action in two cancerous contexts: **(A)** In response to ER stress, in prostate cancer, eIF2α is phosphorylated, via PERK, and activates ATF4, which induces *NIBAN1* expression. *NIBAN1*, in turn, inhibits eIF2α phosphorylation, in a negative feedback limiting cell death. Modified figure from Pällmann et al. (2019). **(B)** In response to ultraviolet ra-diation, *NIBAN1* is phosphorylated, by AKT, in Ser602. pSer602-*NIBAN1* binds to NPM, preventing NPM from binding to MDM2. Thus, free MDM2 interacts with p53, leading to polyubiquitination and its subsequent degradation. Modified figure from Ji et al. (2012).

Ji et al. (2012) demonstrated that NIBAN1 is phosphorylated by AKT in response to ultraviolet stress, in human glioblastoma and lung cancer cell lines, which inhibits cell apoptosis. The suppression of cell death in response to stressful condition has been also associated to *NIBAN1* expression (Ye et al., 2011; Cevik et al., 2020). In prostate cancer, *NIBAN1* is rapidly activated in response to ER stress by ATF4, either by stimulating eIF2α phosphorylation, via PERK, or by inhibiting eIF2α phosphorylation directly, via *NIBAN1* (Figure 2A). These positive and negative feedbacks, in response to ER stress, demonstrate that *NIBAN1* contributes to ATF4 pro-survival role by suppressing senescence and apoptosis, depending on environmental issues and/or cell type (Pällmann et al., 2019). The results are supported by the findings of Cevik et al. (2020) and suggest that *NIBAN1* may be involved in the decision-making process between cell survival and death in response to stressful conditions. In osteoclasts, the *GBF1* knockdown, responsible for activation of the ARFs family and vesicular transport at the endoplasmic reticulum-Golgi interface, led to increased *NIBAN1*, BiP, p-PERK, and p-EIF2α expression (Wen et al., 2021). These genes are considered biomarkers of reticulum stress and their up-regulation seems to promote the autophagic axis of Beclin1, Atg7, p62, and LC3 (Wen et al., 2021).

### Cell Migration and Proliferation

Feng et al. (2019) demonstrated that NIBAN1 interacts with the TIMP-1/MMP2/MMP9 pathway, interfering with the processes of cell migration, invasion and proliferation, in clear cell renal carcinoma (ccRCC), the most aggressive subtype of renal cancer, contributing to tumor progression. Zhang N. et al. (2019) demonstrated that NIBAN1 seems to up-regulate MMP2 and Cyclin D1, inducing cell proliferation and invasion, in the development and progression of non-small cell lung cancer. These findings have been corroborated in both colon cancer and thyroid carcinoma cell lines, whose results showed that decreased *NIBAN1* expression inhibits cell proliferation and migration (Carvalheira et al., 2015; Yuki et al., 2015).

### Cell Apoptosis

As aforementioned, *NIBAN1* is strictly relate to cell death and survival. In fact, pSer602-NIBAN1 by AKT, in response to ultraviolet stress (Figure 2B) showed that phosphorylated NIBAN1 binds to NPM, preventing NPM from binding to MDM2. The resulting free MDM2 interacts with p53, leading to p53 degradation, which in turn inhibits UV-irradiation induced cell apoptosis (Ji et al., 2012). The results indicate that pSer602-NIBAN1 is sufficient to lead to p53 degradation, regulating cell apoptosis (Ji et al., 2012). On the other hand, in head and neck tumors it was observed that the loss of transcriptional activity of p53 was inversely proportional to the expression of *NIBAN1* (Van der Vorst et al., 2012). These findings suggest that *NIBAN1*, in some cancers, may favor cell survival in relation to other stress-dependent manners, regulating apoptosis via p53-independent pathways (Ji et al., 2012). Feng et al. (2019) demonstrated that NIBAN1 seems to play an important role in down-regulation of apoptosis due to its interaction with the MDM2/p53/Bcl2/Bax pathway. *NIBAN1* overexpression induced an increase in MDM2 and Bc12 while decreasing p53 and Bax, responsible for cell death. It had been demonstrated that *NIBAN1* silencing leads to increased apoptotic levels in follicular and papillary thyroid carcinomas cell lines (Carvalheira et al., 2015). These findings suggest that *NIBAN1* may indeed be involved in apoptosis.

Although the comprehension about the role of *NIBAN1* in other pathologies concerning the apoptosis pathway is scarce, it had been shown its participation in tissue damage recovery (Luo et al., 2017; Yim et al., 2020). Vascular injury leads to cell membrane rupture, ATP release, and endothelial dysfunction (Yim et al., 2020). In saphenous vein grafts used for revascularization procedures, the injuries caused during the collection and preparation of the graft lead to failures in the regeneration process, increasing the rates of apoptosis in the implanted vein (Conte et al., 2006). In vascular lesions, the p38/MAPK/AKT pathway inhibits apoptosis through NIBAN1 phosphorylation at serine 602 (Luo et al., 2017; Yim et al., 2020). These findings corroborate with the fact that *NIBAN1* may represent an endogenous regulator of p38 MAPK activation, inhibiting apoptosis in vascular lesions.

**FIGURE 3:**
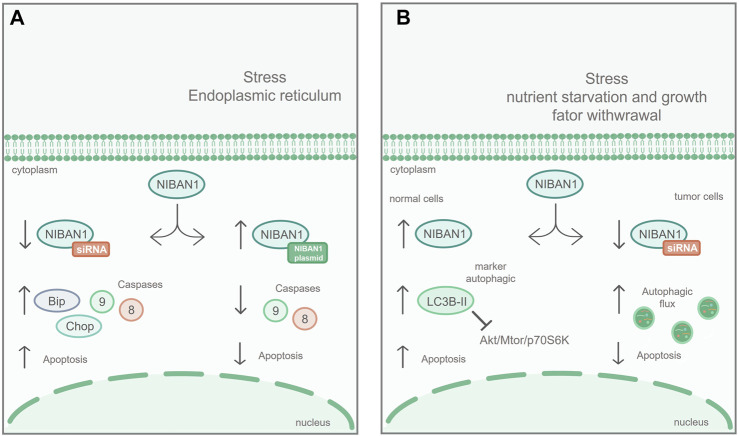
*NIBAN1* in the regulation of apoptosis and autophagy: **(A)** Apoptosis: In renal cell lines, *NIBAN1* silencing increases stress-induced apoptosis of the ER and promotes the expression of caspase 8, caspase 9, Bip, and Chop. On the other hand, overexpression of *NIBAN1* reduces both stress-induced apoptosis and the expression of caspase 8 and caspase 9, suggesting that *NIBAN1* may be involved in the regulation of apoptosis via caspase-dependent pathway. Modified figure from Tang et al. (2019). **(B)** Under nutritional restriction, in normal thyroid cell line, *NIBAN1* expression is increased, together with LC3B-II, an autophagy marker, inhibiting the AKT/mTOR/p70S6K pathway, and increasing cell death. On the other hand, with *NIBAN1* silencing, in thyroid cancer cell line, the autophagic flux is increased, suggesting that *NIBAN1* can inhibit autophagy in these models, avoiding programmed cell death. Modified figure from Nozima et al. (2019).

It has also been described that *NIBAN1* plays an important role in renal interstitial fibrosis, regulating the apoptosis of renal tubular epithelial cells (HK-2) *in vitro* (Tang et al., 2019). Using the unilateral ureteral obstruction (UUO) model, in C57BL/6 mice, it was demonstrated that, during the obstruction development, Niban1 expression gradually decreases while apoptosis rates increase (Tang et al., 2019). Furthermore, *NIBAN1* silencing, in the HK-2 lineage, increased stress-induced apoptosis, promoting caspases 8 and 9 expression, as a markers of apoptosis process, as well as increased the stress biomarkers BIP and CHOP, in ER (Figure 3A). On the other hand, overexpression of *NIBAN1* reduced apoptosis and expression of caspases 8 and 9, as a result of ER stress (Tang et al., 2019). These data suggest that *NIBAN1* seems to be involved in the apoptosis regulation, via a caspase-dependent pathway.

### Cell Autophagy

Nozima et al. (2019), using normal thyroid cell lines derived from human and rat, demonstrated that *NIBAN1* expression was induced along with proteins related to autophagy suppression (Figure 3B) under stress conditions, through AKT/mTOR/p70S6K inhibition pathway. On the other hand, in thyroid cancer cell lines, *NIBAN1* silencing increased autophagic flux, suggesting that *NIBAN1* inhibits autophagy during the thyroid carcinogenic process. These data suggest that *NIBAN1* may play a dual role in the autophagy regulation: 1) It can increase baseline autophagy in normal thyroid cells in response to nutrient and growth factors depletion, and 2) can inhibit autophagy in thyroid carcinomas in the presence of activating mutations in oncogenes (Nozima et al., 2019). These findings are supported by studies with neuronal cell lines in response to treatment with unconjugated bilirubin (UCB), which triggers oxidative stress, through calcium signaling and stress in the ER (Qaisiya et al., 2017). In these cells, treatment with UCB changed the expression of *NIBAN1* along with other proteins related to autophagy (Qaisiya et al., 2017). All these functional studies suggest that *NIBAN1* can be activated early in response to cellular stress, promoting the autophagic process.

### Immune Microenvironment

*NIBAN1* also seems to play important roles in the immune mechanism (Wang et al., 2020; Zhang et al., 2020; Chen et al., 2021; Tan et al., 2021). In colorectal cancer, four-gene signature (*NIBAN1*, *BCL2*, *PMAIP1*, and *RPS6*) have been detected, correlating with immune infiltration (Tan et al., 2021). Zhang et al. (2020) evaluated the transcriptomic heterogeneity of regulatory T cells (Treg) in different tissues: spleen (s-Treg), lymph nodes (LN-Treg), intestine (int-Treg) and visceral adipose tissue (VAT-Treg). They emphasize how the biological context of the tissue is associated with transcriptomic remodeling of Treg cells. They characterized Treg heterogeneity by examining genes signature and demonstrated that elevated levels of 11 gene were present in these tissues, being *NIBAN1* one of them. These results suggest that these genes may play major roles in immune environments (Zhang et al., 2020). The relationship between *NIBAN1* and tumor immune microenvironment may help to understand the molecular mechanisms by which immune escapes are originated. These biological events are crucial processes in tumorigenesis as well as may involve both recruitment of immunosuppressive cells, such as T reg cells, and/or the response to pathogens (Togashi et al., 2019).

## Conclusion

Considering the cell molecular machinery, we emphasize the importance to expand knowledge about the gene functional status in different types of cellular contexts, including diseases and tumorigenesis. The study of gene functions is essential to understand the molecular mechanisms involved in the cellular microenvironment. In this sense, the comprehension of the *NIBAN1* function, in many diseases and cancer progression, is fundamental for the management of cell stability as well as both diseases and cancer treatments.

The increased *NIBAN1* expression in early tumorigenesis, highlights the significance of this gene in cellular survival. This high expression seems to be associated with stress response in different biological conditions (Sun et al., 2007; Ji et al., 2012; Nozima et al., 2019; Pällmann et al., 2019). In these stressful environments, *NIBAN1* downregulates apoptosis to allow the cell survival (Ji et al., 2012; Carvalheira et al., 2015; Feng et al., 2019; Yim et al., 2020). Furthermore, *NIBAN1* plays an ambiguous role in relation to apoptosis and autophagy, and the cellular microenvironment is crucial for its performance in these functions (Qaisiya et al., 2017; Nozima et al., 2019). It had been demonstrated that *NIBAN1* may be involved with protein translation processes to alleviate the ER stress (Sun et al., 2007; Pällmann et al., 2019). *NIBAN1* expression also seems to be associated with the immune microenvironment (Wang et al., 2020; Zhang et al., 2020; Chen et al., 2021; Tan et al., 2021). It is known that the immune microenvironment of the tumor greatly determines the therapeutic effectiveness of immunological cancer treatments (Tang et al., 2021). The *NIBAN1* is part of gene signatures, such as immune infiltration (Tan et al., 2021) and in Treg cells (Zhang et al., 2020). Evidence has demonstrated the importance of tumor infiltration in immune cells, such as tumor-associated macrophages and dendritic cells, T helper and cytotoxic T, in tumor progression, therapeutic responses and prognosis (Liu et al., 2020). The overexpression of *NIBAN1* in regulatory T cells also has an important correlation, this cell type mainly migrates to inflammatory sites and suppresses several types of effector lymphocytes (Togashi et al., 2019). In fact, in the cancer context, T reg cells are often detected in inflamed tumors. Cancers exploit different immune escape mechanisms that are sometimes dependent on certain intrinsic tumor factors, such as mutations in the driver genes. Thus, the signals provided by these mutations may allow tumor cells to recruit T reg cells, mainly by changing the local environment of chemokines and the metabolic environment. Therefore, alterations in the driver genes may play crucial roles in promoting not only tumor growth but also in evading anti-tumor immunity. Thus, understanding the genetic context and how it influences *NIBAN1* expression and the immune environment will yield important results for the understanding of this gene functions (Togashi et al., 2019). Additionally, some studies describe the association of *NIBAN1* with cell proliferation and migration (Carvalheira et al., 2015; Yuki et al., 2015; Feng et al., 2019; Zhang N. et al., 2019). All these data demonstrate the *NIBAN1* roles in cell survival, and consequently, its biological functions associated with tumor progression. Furthermore, pos-translational modifications in *NIBAN1* protein—especially serine 602 phosphorylation—seem to be crucial for its functional activity in apoptosis regulation (Ji et al., 2012; Yim et al., 2020).

Although *NIBAN1* has been described more than 20 years ago, its roles in cancer and other cellular contexts still need to be elucidated. The robust study gathered here demonstrates the importance of *NIBAN1* for cell stability and survival. However, there is much to understand about its broad functionality as well as its role in diagnosis (Adachi et al., 2004; Cerutti et al., 2004; Maciel et al., 2005; Cerutti et al., 2006; Matsumoto et al., 2006; Sun et al., 2007; Ito et al., 2010; Cerutti 2011; Patel et al., 2011; Carvalheira et al., 2013) and therapeutic use (Luo et al., 2017; Yim et al., 2020).

## References

[B1] Adachi H. Majima S. Kon S. Kobayashi T. Kajino K. Mitani H. (2004). Niban Gene is Commonly Expressed in the Renal Tumors: A New Candidate Marker for Renal Carcinogenesis. Oncogene 23 (19), 3495–3500. 10.1038/sj.onc.1207468 14990989

[B2] Boyd R. S. Adam P. J. Patel S. Loader J. A. Berry J. Redpath N. T. (2003). Proteomic Analysis of the Cell-Surface Membrane in Chronic Lymphocytic Leukemia: Identification of Two Novel Proteins, BCNP1 and MIG2B. Leukemia 17 (8), 1605–1612. 10.1038/sj.leu.2402993 12886250

[B3] Braschi B. Denny P. Gray K. Jones T. Seal R. Tweedie S. (2019). Genenames.org: the HGNC and VGNC Resources in 2019. Nucleic Acids Res. 47 (D1), D786–D792. 10.1093/nar/gky930 30304474 PMC6324057

[B4] Cao X. (2016). Self-Regulation and Cross-Regulation of Pattern-Recognition Receptor Signalling in Health and Disease. Nat. Rev. Immunol. 16 (1), 35–50. 10.1038/nri.2015.8 26711677

[B5] Carvalheira G. M. Nozima B. H. N. Riggins G. J. Cerutti J. M. (2013). DDIT3, STT3A (ITM1), ARG2 and FAM129A (Niban, C1orf24) in Diagnosing Thyroid Carcinoma: Variables that May Affect the Performance of This Antibody-Based Test and Promise. Mod. Pathol. 26 (4), 611–613. 10.1038/modpathol.2012.212 23542525

[B6] Carvalheira G. Nozima B. H. Cerutti J. M. (2015). microRNA-106b-Mediated Down-Regulation of C1orf24 Expression Induces Apoptosis and Suppresses Invasion of Thyroid Cancer. Oncotarget 6 (29), 28357–28370. 10.18632/oncotarget.4947 26317551 PMC4695065

[B7] Cerutti J. M. (2011). Employing Genetic Markers to Improve Diagnosis of Thyroid Tumor fine Needle Biopsy. Curr. Genomics 12 (8), 589–596. 10.2174/138920211798120781 22654558 PMC3271311

[B8] Cerutti J. M. Delcelo R. Amadei M. J. Nakabashi C. Maciel R. M. B. Peterson B. (2004). A Preoperative Diagnostic Test that Distinguishes Benign from Malignant Thyroid Carcinoma Based on Gene Expression. J. Clin. Invest. 113 (8), 1234–1242. 10.1172/JCI19617 15085203 PMC385398

[B9] Cerutti J. M. Latini F. R. M. Nakabashi C. Delcelo R. Andrade V. P. Amadei M. J. (2006). Diagnosis of Suspicious Thyroid Nodules Using Four Protein Biomarkers. Clin. Cancer Res. 12 (11 Pt 1), 3311–3318. 10.1158/1078-0432.CCR-05-2226 16740752

[B10] Cevik M. Gunduz M. K. Deliorman G. Susleyici B. (2020). Alterations in Niban Gene Expression as a Response to Stress Conditions in 3T3-L1 Adipocytes. Mol. Biol. Rep. 47 (12), 9399–9408. 10.1007/s11033-020-05992-5 33185830

[B11] Chang H. H. Y. Pannunzio N. R. Adachi N. Lieber M. R. (2017). Non-Homologous DNA End Joining and Alternative Pathways to Double-Strand Break Repair. Nat. Rev. Mol. Cell Biol 18 (8), 495–506. 10.1038/nrm.2017.48 28512351 PMC7062608

[B12] Chen S. Evans H. G. Evans D. R. (2011). FAM129B/MINERVA, a Novel Adherens junction-associated Protein, Suppresses Apoptosis in HeLa Cells. J. Biol. Chem. 286 (12), 10201–10209. 10.1074/jbc.M110.175273 21148485 PMC3060473

[B13] Chen S. Yang D. Lei C. Li Y. Sun X. Chen M. (2019). Identification of Crucial Genes in Abdominal Aortic Aneurysm by WGCNA. PeerJ 7, e7873. 10.7717/peerj.7873 31608184 PMC6788446

[B14] Chen X. Cubillos-Ruiz J. R. (2021). Endoplasmic Reticulum Stress Signals in the Tumour and its Microenvironment. Nat. Rev. Cancer 21 (2), 71–88. 10.1038/s41568-020-00312-2 33214692 PMC7927882

[B15] Chen Y. Zhu S. Pei Y. Hu J. Hu Z. Liu X. (2021). Differential microRNA Expression in Newcastle Disease Virus-Infected HeLa Cells and its Role in Regulating Virus Replication. Front. Oncol. 11, 616809. 10.3389/fonc.2021.616809 34150610 PMC8211993

[B16] Conte M. S. Bandyk D. F. Clowes A. W. Moneta G. L. Seely L. Lorenz T. J. (2006). Results of PREVENT III: A Multicenter, Randomized Trial of Edifoligide for the Prevention of Vein Graft Failure in Lower Extremity Bypass Surgery. J. Vasc. Surg. 43 (4), 742–751. 10.1016/j.jvs.2005.12.058 16616230

[B17] Evstafieva A. G. Kovaleva I. E. Shoshinova M. S. Budanov A. V. Chumakov P. M. (2018). Implication of KRT16, FAM129A and HKDC1 Genes as ATF4 Regulated Components of the Integrated Stress Response. PLoS One 13 (2), e0191107. 10.1371/journal.pone.0191107 29420561 PMC5805170

[B18] Feng X. Yan N. Sun W. Zheng S. Jiang S. Wang J. (2019). miR-4521-FAM129A Axial Regulation on ccRCC Progression Through TIMP-1/MMP2/MMP9 and MDM2/p53/Bcl2/Bax Pathways. Cell Death Discov. 5, 89. 10.1038/s41420-019-0167-5 31016032 PMC6465337

[B19] Fuchs Y. Steller H. (2015). Live to Die Another Way: Modes of Programmed Cell Death and the Signals Emanating from Dying Cells. Nat. Rev. Mol. Cell Biol. 16 (6), 329–344. 10.1038/nrm3999 25991373 PMC4511109

[B20] Galluzzi L. Baehrecke E. H. Ballabio A. Boya P. Bravo‐San Pedro J. M. Cecconi F. (2017). Molecular Definitions of Autophagy and Related Processes. EMBO J. 36 (13), 1811–1836. 10.15252/embj.201796697 28596378 PMC5494474

[B21] Galluzzi L. Yamazaki T. Kroemer G. (2018). Linking Cellular Stress Responses to Systemic Homeostasis. Nat. Rev. Mol. Cell Biol 19 (11), 731–745. 10.1038/s41580-018-0068-0 30305710

[B22] Hetz C. Papa F. R. (2018). The Unfolded Protein Response and Cell Fate Control. Mol. Cell 69 (2), 169–181. 10.1016/j.molcel.2017.06.017 29107536

[B23] Hino O. (2004). Multistep Renal Carcinogenesis in the Eker (Tsc 2 Gene Mutant) Rat Model. Curr. Mol. Med. 4 (8), 807–811. 10.2174/1566524043359692 15579027

[B24] Hornbeck P. V. Zhang B. Murray B. Kornhauser J. M. Latham V. Skrzypek E. (2015). PhosphoSitePlus, 2014: Mutations, PTMs and Recalibrations. Nucleic Acids Res. 43, D512–D520. 10.1093/nar/gku1267 25514926 PMC4383998

[B25] Ito S. Fujii H. Matsumoto T. Abe M. Ikeda K. Hino O. (2009). Frequent Expression of Niban in Head and Neck Squamous Cell Carcinoma and Squamous Dysplasia. Head Neck 32 (1), NA. 10.1002/hed.21153 19536772

[B26] Ji H. Ding Z. Hawke D. Xing D. Jiang B. H. Mills G. B. (2012). AKT‐Dependent Phosphorylation of Niban Regulates Nucleophosmin‐ and MDM2‐Mediated p53 Stability and Cell Apoptosis. EMBO Rep. 13 (6), 554–560. 10.1038/embor.2012.53 22510990 PMC3367238

[B27] Jiang J. Bi Y. Liu X. P. Yu D. Yan X. Yao J. (2020). To Construct a ceRNA Regulatory Network as Prognostic Biomarkers for Bladder Cancer. J. Cell. Mol. Medi 24 (9), 5375–5386. 10.1111/jcmm.15193 PMC7205833 32233022

[B28] Jumper J. Evans R. Pritzel A. Green T. Figurnov M. Ronneberger O. (2021). Highly Accurate Protein Structure Prediction with AlphaFold. Nature 596 (7873), 583–589. 10.1038/s41586-021-03819-2 34265844 PMC8371605

[B29] Kannangai R. Diehl A. M. Sicklick J. Rojkind M. Thomas D. Torbenson M. (2005). Hepatic Angiomyolipoma and Hepatic Stellate Cells Share a Similar Gene Expression Profile. Hum. Pathol. 36 (4), 341–347. 10.1016/j.humpath.2005.01.002 15891994

[B30] Lenoir M. Kufareva I. Abagyan R. Overduin M. (2015). Membrane and Protein Interactions of the Pleckstrin Homology Domain Superfamily. Membranes 5 (4), 646–663. 10.3390/membranes5040646 26512702 PMC4704004

[B31] Liu J. Qin J. Mei W. Zhang H. Yuan Q. Peng Z. (2014). Expression of Niban in Renal Interstitial Fibrosis. Nephrology 19 (8), 479–489. 10.1111/nep.12266 24750539

[B32] Liu R. Hu R. Zeng Y. Zhang W. Zhou H.-H. (2020). Tumour Immune Cell Infiltration and Survival after Platinum-Based Chemotherapy in High-Grade Serous Ovarian Cancer Subtypes: A Gene Expression-Based Computational Study. EBioMedicine 51, 102602. 10.1016/j.ebiom.2019.102602 31911269 PMC6948169

[B33] Luo W. Feldman D. McCallister R. Brophy C. Cheung-Flynn J. (2017). P2X7R Antagonism After Subfailure Overstretch Injury of Blood Vessels Reverses Vasomotor Dysfunction and Prevents Apoptosis. Purinergic Signal. 13 (4), 579–590. 10.1007/s11302-017-9585-0 28905300 PMC5714848

[B34] Maciel R. M. B. Kimura E. T. Cerutti J. M. (2005). Pathogenesis of Differentiated Thyroid Cancer (Papillary and Follicular). Arq Bras Endocrinol. Metab. 49 (5), 691–700. 10.1590/s0004-27302005000500009 16444351

[B35] Majima S. Kajino K. Fukuda T. Otsuka F. Hino O. (2000). A Novel Gene “Niban” Upregulated in Renal Carcinogenesis: Cloning by the cDNA-Amplified Fragment Length Polymorphism Approach. Jpn. J. Cancer Res. 91 (9), 869–874. 10.1111/j.1349-7006.2000.tb01027.x 11011112 PMC5926447

[B36] Matsumoto F. Fujii H. Abe M. Kajino K. Kobayashi T. Matsumoto T. (2006). A Novel Tumor Marker, Niban, is Expressed in Subsets of Thyroid Tumors and Hashimoto's Thyroiditis. Hum. Pathol. 37 (12), 1592–1600. 10.1016/j.humpath.2006.06.022 16949643

[B37] Miller S. Rogers H. A. Lyon P. Rand V. Adamowicz-Brice M. Clifford S. C. (2011). Genome-wide Molecular Characterization of central Nervous System Primitive Neuroectodermal Tumor and Pineoblastoma. Neuro Oncol. 13 (8), 866–879. 10.1093/neuonc/nor070 21798848 PMC3145471

[B38] Nozima B. H. Mendes T. B. Pereira G. J. d. S. Araldi R. P. Iwamura E. S. M. Smaili S. S. (2019). FAM129A Regulates Autophagy in Thyroid Carcinomas in an Oncogene-dependent Manner. Endocr. Relat. Cancer 26 (1), 227–238. 10.1530/ERC-17-0530 30400008

[B39] Old W. M. Shabb J. B. Houel S. Wang H. Couts K. L. Yen C.-y. (2009). Functional Proteomics Identifies Targets of Phosphorylation by B-Raf Signaling in Melanoma. Mol. Cell 34 (1), 115–131. 10.1016/j.molcel.2009.03.007 19362540 PMC2735263

[B40] Pällmann N. Livgård M. Tesikova M. Zeynep Nenseth H. Akkus E. Sikkeland J. (2019). Regulation of the Unfolded Protein Response Through ATF4 and FAM129A in Prostate Cancer. Oncogene 38 (35), 6301–6318. 10.1038/s41388-019-0879-2 31312022

[B41] Patel M. R. Stadler M. E. Deal A. M. Kim H. S. Shores C. G. Zanation A. M. (2011). STT3A, C1orf24, TFF3: Putative Markers for Characterization of Follicular Thyroid Neoplasms from Fine-Needle Aspirates. Laryngoscope 121 (5), 983–989. 10.1002/lary.21736 21520112

[B42] Qaisiya M. Mardešić P. Pastore B. Tiribelli C. Bellarosa C. (2017). The Activation of Autophagy Protects Neurons and Astrocytes Against Bilirubin-Induced Cytotoxicity. Neurosci. Lett. 661, 96–103. 10.1016/j.neulet.2017.09.056 28965934

[B43] Ralli M. Angeletti D. Fiore M. D’Aguanno V. Lambiase A. Artico M. (2020). Hashimoto’s Thyroiditis: An Update on Pathogenic Mechanisms, Diagnostic Protocols, Therapeutic Strategies, and Potential Malignant Transformation. Autoimmun. Rev. 19 (10), 102649. 10.1016/j.autrev.2020.102649 32805423

[B44] Richter K. Haslbeck M. Buchner J. (2010). The Heat Shock Response: Life on the Verge of Death. Mol. Cell 40 (2), 253–266. 10.1016/j.molcel.2010.10.006 20965420

[B45] Salgado-Albarrán M. González-Barrios R. Guerra-Calderas L. Alcaraz N. Estefanía Sánchez-Correa T. Castro-Hernández C. (2019). The Epigenetic Factor BORIS (CTCFL) Controls the Androgen Receptor Regulatory Network in Ovarian Cancer. Oncogenesis 8 (8), 41. 10.1038/s41389-019-0150-2 31406110 PMC6690894

[B46] Shaw G. L. Whitaker H. Corcoran M. Dunning M. J. Luxton H. Kay J. (2016). The Early Effects of Rapid Androgen Deprivation on Human Prostate Cancer. Eur. Urol. 70 (2), 214–218. 10.1016/j.eururo.2015.10.042 26572708 PMC4926724

[B47] Sun G. D. Kobayashi T. Abe M. Tada N. Adachi H. Shiota A. (2007). The Endoplasmic Reticulum Stress-Inducible Protein Niban Regulates eIF2α and S6K1/4E-BP1 Phosphorylation. Biochem. Biophys. Res. Commun. 360 (1), 181–187. 10.1016/j.bbrc.2007.06.021 17588536

[B48] Suomalainen A. Battersby B. J. (2018). Mitochondrial Diseases: The Contribution of Organelle Stress Responses to Pathology. Nat. Rev. Mol. Cell Biol. 19 (2), 77–92. 10.1038/nrm.2017.66 28792006

[B49] Tan X. Mao L. Huang C. Yang W. Guo J. Chen Z. (2021). Comprehensive Analysis of lncRNA-miRNA-mRNA Regulatory Networks for Microbiota-Mediated Colorectal Cancer Associated with Immune Cell Infiltration. Bioengineered 12 (1), 3410–3425. 10.1080/21655979.2021.1940614 34227920 PMC8806860

[B50] Tang S. Wang J. Liu J. Huang Y. Zhou Y. Yang S. (2019). Niban Protein Regulates Apoptosis in HK-2 Cells via Caspase-Dependent Pathway. Ren. Fail. 41 (1), 455–466. 10.1080/0886022X.2019.1619582 31163002 PMC6566711

[B51] Tang T. Huang X. Zhang G. Hong Z. Bai X. Liang T. (2021). Advantages of Targeting the Tumor Immune Microenvironment over Blocking Immune Checkpoint in Cancer Immunotherapy. Sig Transduct. Target. Ther. 6 (1), 72. 10.1038/s41392-020-00449-4 PMC7896069 33608497

[B52] Thomas B. C. Kay J. D. Menon S. Vowler S. L. Dawson S. N. Bucklow L. J. (2016). Whole Blood mRNA in Prostate Cancer Reveals a Four-Gene Androgen Regulated Panel. Endocr. Relat. Cancer 23 (10), 797–812. 10.1530/ERC-16-0287 27578825

[B53] Togashi Y. Shitara K. Nishikawa H. (2019). Regulatory T Cells in Cancer Immunosuppression - Implications for Anticancer Therapy. Nat. Rev. Clin. Oncol. 16 (6), 356–371. 10.1038/s41571-019-0175-7 30705439

[B54] Uhlén M. Björling E. Agaton C. Szigyarto C. A.-K. Amini B. Andersen E. (2005). A Human Protein Atlas for normal and Cancer Tissues Based on Antibody Proteomics. Mol. Cell Proteom. 4 (12), 1920–1932. 10.1074/mcp.M500279-MCP200 16127175

[B55] Uhlén M. Fagerberg L. Hallström B. M. Lindskog C. Oksvold P. Mardinoglu A. (2015). Tissue-Based Map of the Human Proteome. Science 347 (6220), 1260419. 10.1126/science.1260419 25613900

[B56] Uhlen M. Zhang C. Lee S. Sjöstedt E. Fagerberg L. Bidkhori G. (2017). A Pathology Atlas of the Human Cancer Transcriptome. Science 357 (6352). 10.1126/science.aan2507 28818916

[B57] Van der Vorst S. Dekairelle A.-F. Weynand B. Hamoir M. Gala J.-L. (2012). Assessment of p53 Functional Activity in Tumor Cells and Histologically Normal Mucosa from Patients with Head and Neck Squamous Cell Carcinoma. Head Neck 34 (11), 1542–1550. 10.1002/hed.21960 22109999

[B58] Varadi M. Anyango S. Deshpande M. Nair S. Natassia C. Yordanova G. (2022). AlphaFold Protein Structure Database: Massively Expanding the Structural Coverage of Protein-Sequence Space with High-Accuracy Models. Nucleic Acids Res. 50 (D1), D439–D444. 10.1093/nar/gkab1061 34791371 PMC8728224

[B59] Vihervaara A. Duarte F. M. Lis J. T. (2018). Molecular Mechanisms Driving Transcriptional Stress Responses. Nat. Rev. Genet. 19 (6), 385–397. 10.1038/s41576-018-0001-6 29556092 PMC6036639

[B60] Walter P. Ron D. (2011). The Unfolded Protein Response: From Stress Pathway to Homeostatic Regulation. Science 334 (6059), 1081–1086. 10.1126/science.1209038 22116877

[B61] Wang C. Shi Z. Zhang Y. Li M. Zhu J. Huang Z. (2021). CBFβ Promotes Colorectal Cancer Progression through Transcriptionally Activating OPN, FAM129A, and UPP1 in a RUNX2-Dependent Manner. Cell Death Differ. 28, 3176–3192. 10.1038/s41418-021-00810-2 34050318 PMC8563980

[B62] Wang X. Duanmu J. Fu X. Li T. Jiang Q. (2020). Analyzing and Validating the Prognostic Value and Mechanism of colon Cancer Immune Microenvironment. J. Transl. Med. 18 (1), 324. 10.1186/s12967-020-02491-w 32859214 PMC7456375

[B63] Wen C. Zhou Y. Xu Y. Tan H. Pang C. Liu H. (2021). The Regulatory Role of GBF1 on Osteoclast Activation Through EIF2a Mediated ER Stress and Novel Marker FAM129A Induction. Front. Cell Dev. Biol. 9, 706768. 10.3389/fcell.2021.706768 34513838 PMC8424197

[B64] Ye F. Zhang H. Yang Y.-X. Hu H.-D. Sze S. K. Meng W. (2011). Comparative Proteome Analysis of 3T3-L1 Adipocyte Differentiation Using iTRAQ-Coupled 2D LC-MS/MS. J. Cell. Biochem. 112 (10), 3002–3014. 10.1002/jcb.23223 21678470

[B65] Yick C. Y. Zwinderman A. H. Kunst P. W. Grünberg K. Mauad T. Chowdhury S. (2014). Gene Expression Profiling of Laser Microdissected Airway Smooth Muscle Tissue in Asthma and Atopy. Allergy 69 (9), 1233–1240. 10.1111/all.12452 24888725

[B66] Yick C. Y. Zwinderman A. H. Kunst P. W. Grünberg K. Mauad T. Fluiter K. (2013). Glucocorticoid-induced Changes in Gene Expression of Airway Smooth Muscle in Patients with Asthma. Am. J. Respir. Crit. Care Med. 187 (10), 1076–1084. 10.1164/rccm.201210-1886OC 23491407

[B67] Yim T. W. Perling D. Polcz M. Komalavilas P. Brophy C. Cheung‐Flynn J. (2020). A Cell Permeant Phosphopeptide Mimetic of Niban Inhibits p38 MAPK and Restores Endothelial Function After Injury. FASEB J. 34 (7), 9180–9191. 10.1096/fj.201902745R 32396246 PMC7383822

[B68] Yuki R. Aoyama K. Kubota S. Yamaguchi N. Kubota S. Hasegawa H. (2015). Overexpression of Zinc‐Finger Protein 777 (ZNF777) Inhibits Proliferation at Low Cell Density Through Down‐Regulation of FAM129A. J. Cell. Biochem. 116 (6), 954–968. 10.1002/jcb.25046 25560148

[B69] Zhang K. Yu S.-S. Li G.-Y. He L. Liang X.-Q. (2019). miR-135a Deficiency Inhibits the AR42J Cells Damage in Cerulein-Induced Acute Pancreatitis through Targeting FAM129A. Pflugers Arch. 471 (11–12), 1519–1527. 10.1007/s00424-019-02329-5 31729558

[B70] Zhang N. Zhou X. M. Yang F. F. Zhang Q. Miao Y. Hou G. (2019). FAM129A Promotes Invasion and Proliferation by Activating FAK Signaling Pathway in Non-small Cell Lung Cancer. Int. J. Clin. Exp. Pathol. 12 (3), 893–900. 31933898 PMC6945167

[B71] Zhang R. Xu K. Shao Y. Sun Y. Saredy J. Cutler E. (2020). Tissue Treg Secretomes and Transcription Factors Shared with Stem Cells Contribute to a Treg Niche to Maintain Treg-Ness With 80% Innate Immune Pathways, and Functions of Immunosuppression and Tissue Repair. Front. Immunol. 11, 632239. 10.3389/fimmu.2020.632239 33613572 PMC7892453

[B72] Zhu N. Hou J. Wu Y. Liu J. Li G. Zhao W. (2018). Integrated Analysis of a Competing Endogenous RNA Network Reveals Key lncRNAs as Potential Prognostic Biomarkers for Human Bladder Cancer. Medicine 97 (35), e11887. 10.1097/MD.0000000000011887 30170380 PMC6392549

